# Methods of Formalin Disinfection

**Published:** 1904-03

**Authors:** 


### Methods of Formalin Disinfection.

Sembritzki, of Königsberg (*Therap. Monatshefte,* November, 1903,) says that formalin is most effectively and conveniently used as pastils vaporised in the formalin lamp. Its employment effects both the inhibition of germ growth and deodorisation. It rapidly destroys the bad odor of waiting-, sick-, bedrooms and nurseries. It is wholly free from fire danger. The flame should be turned so low that but one pastil is vaporised in the course of a night. This causes no discomfort whatever. The author is greatly pleased with the action of the formalin lamp, not only in infectious diseases, but especially in affections of the respiratory organs, where air purification is essential, as in coryza, bronchial catarrhs, influenza, phthisis, and the like.

Especially brilliant results has the lamp given in whooping cough. The toxin of pertussis is still unknown, though its specific germ has been insolated; but it is doubtless present in the breath and sputum. Efforts have long been made to sterilise the air by ventilation, phenol, etc. Here formalin vaporisation renders the air antiseptic as well as aseptic. The author prescribes it for every case of pertussis and always has had excellent results; often the disease is cut short and generally it is rendered much shorter and milder. Similar experiences have already been recorded by Dr. Jezdik, of Prague (Zeitung der böhmischen Aerzte, September 14, 1901,) and Dr. Lamallerée, Varennes (Revue Mensuelle des maladies de l'Enfance, February, 1902,). If the vaporisation is carefully controlled, no irritation will be set up. The lamp should contain two or three pastils, the flame being kept very low. It is placed in the sickroom or in an adjoining apartment. The room in which the patient is kept should be exchanged every day and the one not used should be well aired.

